# The Interpretation of Graphical Information in Word Processing

**DOI:** 10.3390/e24101492

**Published:** 2022-10-19

**Authors:** Mária Csernoch, János Máth, Tímea Nagy

**Affiliations:** 1 Faculty of Informatics, University of Debrecen, Kassai út 26, 4028 Debrecen, Hungary; 2 Institute of Psychology, Faculty of Humanities, University of Debrecen, Egyetem tér 1, 4032 Debrecen, Hungary

**Keywords:** word processing, cursor, automated numbering, GUI, entropy, redundancy

## Abstract

Word processing is one of the most popular digital activities. Despite its popularity, it is haunted by false assumptions, misconceptions, and ineffective and inefficient practices leading to erroneous digital text-based documents. The focus of the present paper is automated numbering and distinguishing between manual and automated numbering. In general, one bit of information on the GUI—the position of the cursor—is enough to tell whether a numbering is manual or automated. To decide how much information must be put on the channel—the teaching–learning process—in order to reach end-users, we designed and implemented a method that includes the analysis of teaching, learning, tutorial, and testing sources, the collection and analysis of Word documents shared on the internet or in closed groups, the testing of grade 7–10 students’ knowledge in automated numbering, and calculating the entropy of automated numbering. The combination of the test results and the semantics of the automated numbering was used to measure the entropy of automated numbering. It was found that to transfer one bit of information on the GUI, at least three bits of information must be transferred during the teaching–learning process. Furthermore, it was revealed that the information connected to numbering is not the pure use of tools, but the semantics of this feature put into a real-world context.

## 1. Introduction

Word processing is one of the most popular but contradictory end-user activities. The popularity is obvious because almost everyone who has access to computers uses word processor applications [1,2]. However, the quality of output documents calls into question the effectiveness of the word-processing processes and the use of Graphical User Interfaces (GI) [2,3,4,5,6,7,8,9,10,11,12,13,14,15,16,17,18,19]. It is said that “Fundamentally, we claim that every EUD [End User Development] system should attempt to keep the learning challenges in proportion to the skills end-users have. By adopting this perspective, EUD can actively scaffold a process during which end-users pick up new EUD tools and gradually learn about new functionality” [19]. “In short, the significant advantages of word processing are available exclusively to those who are proficient in the use of the hardware and software; they will be inaccessible to those who have only a little understanding of word processing. A stand-alone computer skills course (taught by a school or by a computer dealer) may not be the best means to teach substantial knowledge of word processing; examples and practice will inevitably be simulated and artificial, and there will be little motivation to fully understand the applications” [1].

### 1.1. Aims and Methods

In the present paper, we aim to set up a method to measure the entropy of graphical data presented in word processing interfaces (GUI) and the information which end-users can draw on and utilize in their process of handling automated numbering in text-based documents.

**Figure 1:**
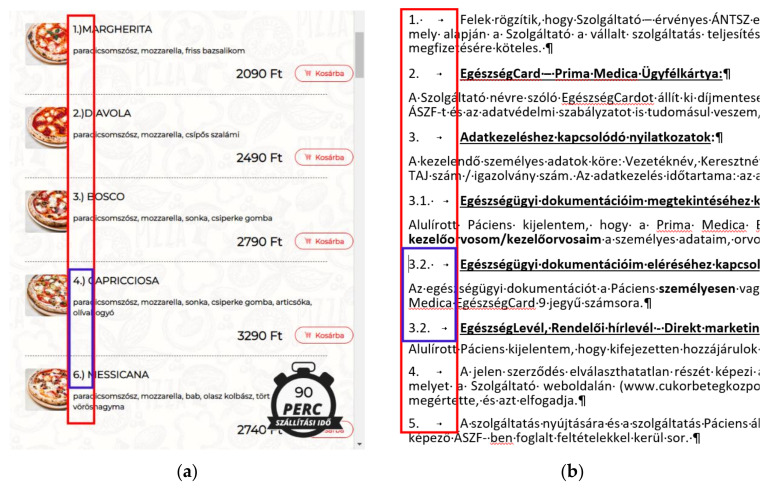
A gap in the manual numbering of a webpage (**a**) and a duplication of numbering in a word-processed document (**b**).

For the present study, automated numbering is selected because it is one of the most widely used algorithm-driven features of digital texts. It is a feature whose misuse can result in gaping lists (broken sequence of numbers) (Figure 1a), duplication in numbering (Figure 1b), and serious financial losses in both the creation and the modification phases of the document. Furthermore, this is one of the features which clearly distinguishes typewriters from word processors/presentation editors/webpage editors due to the algorithms behind them.

In advance of the statistical analysis, we set up an intuitive measuring system based on the semantics and GUI elements of automated numbering. Following this analysis, we calculate the entropy and the redundancy which would provide an objective measuring system to teach automated numbering beyond the mere use of the tool. To calculate the entropy, the results of the mini competence test are used [20] with a sample of 8517 Hungarian students from grades 7–10 (further details are in Section 2).

The calculated entropy would provide an objective measurement of the minimum redundancy to reach students/end-users with the data put on the GUI, which is crucial for the effective use of GUI-based word processing.

With this method, we can find ways to express those features which are beyond the simple use of tools but allow us to reveal the algorithms of various word-processing features.

### 1.2. Hypothesis

Using the graphical and calculated data from the GUI and the mini competence test, respectively, we formulated our hypothesis. The comparison of the two values would reveal how much data must be put in the channel in the teaching–learning process to make students understand the graphical messages and use them effectively and efficiently to solve real-world problems.

Our hypothesis is the following:

Based on the semantics of the automated numbering, three bits of data must be put on the channel to reach end-users with little understanding. Calculating the entropy of automated numbering would prove this content-based value.

### 1.3. The Algorithm of the Process

The goal of the present paper is to obtain to essence of the errors in a natural-language word-processed text where debugging tools are not, or only scarcely, available. Unlike in artificial languages, word processors do not provide any suggestions for debugging and/or discussing. Consequently, we must find other approaches to handle and measure how correct or erroneous digital natural language texts are. In the present paper, one feature of word processing, namely the automated numbering, is selected for discussion.

The idea behind our approach is to measure the information content of the data put on the channel. To achieve our goal, we intend to use the concept of information entropy and find a way to explain how data put on the software interface and in the teaching–learning process reach students.

The process of our research is as follows:

- A mini paper-based competence test was carried out to measure students’ knowledge of automated numbering. One-bit data were presented as the position of the cursor in the example of five samples. Based on this data, students must decide whether the samples are correct or not (automated or manual numbering);
- The analysis of the most popular word processors was carried out with a focus on tools for supporting automated numbering and for displaying non-printing characters;
- The analysis of the semantics of automated numbering was carried out in MS Word. What tools the software offers to complete and modify automated numbering and what graphical data are put on the interface to indicate the presence of numbering were tested;
- We then built a corpus of word-processed texts with DOC or DOCX extensions;
- Considering Shannon’s original definition of entropy and formulating our concept of the entropy of the selected phenomenon (automated numbering), we aimed to measure how much data should be put on the different channels to solve the one-bit problem presented in the mini competence test (detailed in Section 3.3).

Obviously, the structure of the paper does not follow the process of the research since some of the steps were carried out parallel while others are restructured for better understanding.

## 2. Materials and Methods

### 2.1. Selection of Application

To cover all the existing word processing applications is far beyond the scope of the present study. Furthermore, non-printing characters play a crucial role in the analyses of documents because they carry fundamental information (Section 1.3). Consequently, it has been established that the analyses must be carried out in a word processor which is widely used and where displaying the non-printing characters is simple (Section 1.3) [21,22,23,24]. Considering these requirements, Microsoft Word features and documents are analyzed.

### 2.2. Automated Numbering

The focus of the present study is automated numbering, including bullets and multi-level numbering. At this point, we cannot leave unremarked the fact that in both Word and other applications, these three commands are treated as three different features—three buttons are offered to reach them despite the fact that this is not so. The algorithm behind these features is the same, only the leading characters are different. From this point on, all three tools are referred to as numbering.

In advance of this study, we collected Word documents with numbered list(s) to reveal how numbering is carried out, and how consciously automated numbering is applied in Word documents.

Collecting and analyzing Word documents revealed that numbering is a popular feature, and according to teaching, learning, and tutorial materials, is only a one-click simple feature. For the present study, the corpus of 110 documents from various languages was analyzed. The corpus consists of documents collected from the internet (searches launched with filetype:doc and filetype:docx specifications) and from private collections primarily created by students and teachers.

**Figure 2:**
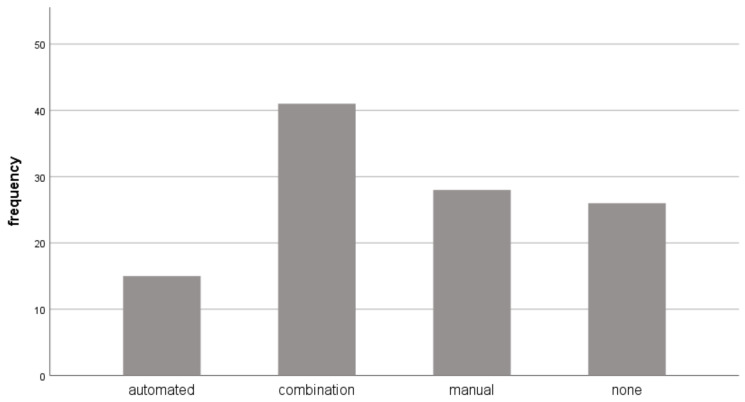
The frequency of documents without numbering (none), using it correctly (automated), creating numbering manually (manual), and using the automated and manual solutions arbitrarily (in combination) considering all the analyzed documents.

The analysis of the documents of the corpus focused only on automated numbering. Figure 2 shows the results of categorization based on the use of numbering. The following four categories were established:

- Documents without numbering (26 documents);
- Automated numbering (15 documents);
- Manual numbering (carried out by typing both the leading and separator character(s)) (28 documents);
- A combination of automated and manual numbering (41 documents);

Of the analyzed documents, approximately three-quarters had numbering. Among those documents which had numbering, 18% used automated numbering while the others used the pure manual form or a combination of automated and manual numbering (82%) (Figure 2). These percentages clearly reveal that numbering is much more demanding than teaching materials claim, and end-users need more information on the proper use of this feature.

Among the 110 Word documents, there are teaching, learning, tutorial, and testing materials connected to word processing. In the following step, these documents were tested. The aim of this testing phase was to reveal how properly these paragons use numbering.

**Figure 3:**
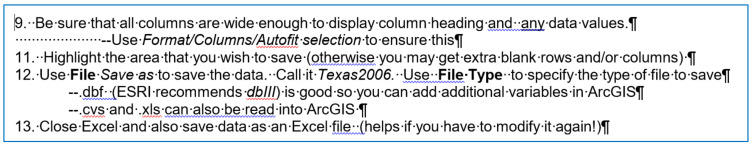
Manual two-level numbering in a testing document.

In Figure 3, the position of the cursor in Line 1 clearly indicates the manual numbering of a testing document, and one of the most common errors in connection to manual numbering—a missing number. Beyond manual numbering, there are other errors in the text:

- Imitation of indentation with Space characters (Line 2);
- Double Space characters in the middle of the sentences (Lines 1, 3, 4, 7);
- Varying number of Space characters following the numbering;
- Arbitrary punctuation;
- Arbitrarily used font styles.

**Figure 4:**
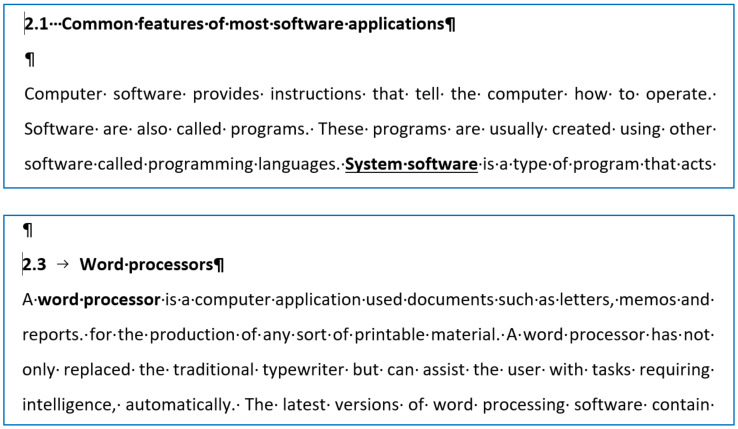
Manual two-level numbering in a teaching document.

Figure 4 shows teaching material whose second-level numbering is manual, which is well documented by the position of the cursor at the beginning of the numbered lines. In the 2.1 example, the number is followed by multiple Space characters, while in the 2.3 example, it is followed by a fake Tabulator character to imitate the automated numbering. The samples contain further errors, which are the following:

- An empty paragraph before and after the numbered paragraphs;
- Inconsistent use of vertical spaces before and after the numbered paragraphs;
- Underlining;
- Unnecessary bold;
- Justified alignment without hyphenation;
- A semantic error. Because software and programs are not synonyms ({programs} ⊂ {software}), the set of software includes the set of programs e.g., data files ∈ {software} but data files ∉ ({programs});
- A semantic error in the paragraph following paragraph 2.3.

**Figure 5:**
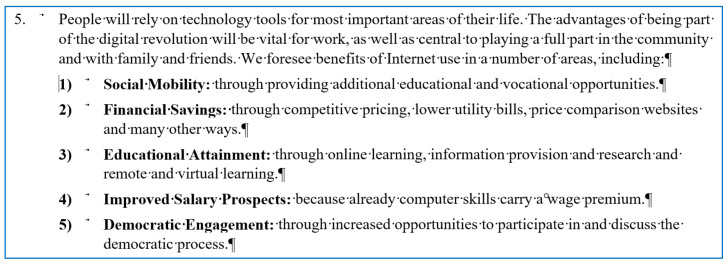
Manual two-level numbering in a report focusing on digital skills.

In Figure 5, a section of a 209 page-long manually numbered document is presented. The cursor is positioned on the second level of numbering which clearly indicates manual numbering. One further error of the document is that, despite the level of numbering, all the paragraphs are numbered with one single number, without referring to the hypernym paragraph(s) (5 and 5 are at both the hypernym and the hyponym levels).

**Figure 6:**
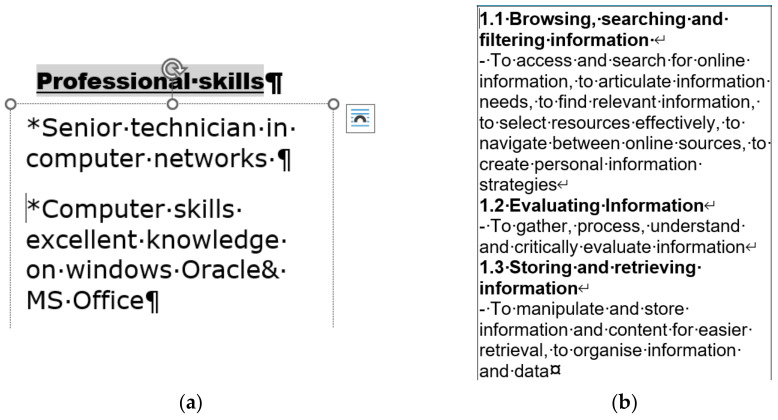
A section from a CV of an informatician applying for a PhD position in informatics (**a**) and a section of teaching material in informatics (**b**).

Figure 6a is a piece selected from a CV whose author claims that he has excellent knowledge of informatics. His self-assessment is based on the MSc degree gained in informatics. Figure 6b is an extract from some teaching material. The position of the cursor clearly indicates manual numbering. Furthermore, the line breaks at the end of the numbered lines—instead of the end-of-paragraph marks—make it clear that, in one paragraph, two-level automated numbering cannot be carried out since numbering is a paragraph-formatting domain.

### 2.3. Testing

A mini competence test was carried out in grades 7–10 all over Hungary [20]. Considering all four grades, the sample size of this analysis is 8517. In the mini competence test, word-processing knowledge was tested by multi-choice questions where each question was accompanied by a screenshot that presented the GUI with a word-processing problem [20]. The questions cover automated numbering, paragraph formats, typographic and syntactic errors, and recognition of sources. For the present study, we selected the task handling automated numbering.

**Figure 7:**
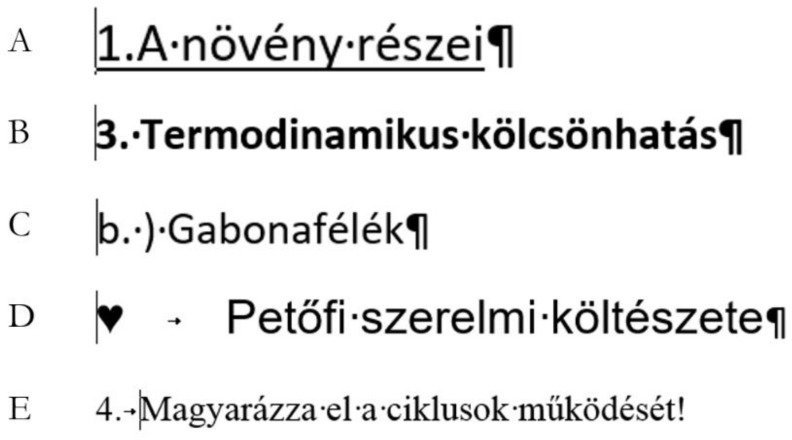
The five samples of numbering presented in a mini competence test [20]. The position of the cursor indicates that samples A–D are manually numbered while E is automated.

The examples presented in Figure 3, Figure 4, Figure 5 and Figure 6 reveal that the position of the cursor clearly indicates whether the numbering is manual or automated. In the first line of a paragraph, the leftmost position of the cursor always shows the first typed character. Since automated numbering is formatting, the cursor cannot be placed to the left of the numbering character. Based on this piece of information—the first position of the cursor—one can tell that Figure 7A–D are manual, and E is automated numbering.

In the present study, the recognition of automated numbering is tested. The question of the task was “Which numbering is correct? Circle the correct answers. (you may mark more than one answer)”, and the samples were presented as shown in Figure 7. The aim of the task is to reveal whether the students know that one bit of information—the position of the cursor—is enough to answer the question. The one bit with its two values matches the two options, namely, whether the cursor can be positioned the furthest to the left in the line of the number/character or not.

**Figure 8:**
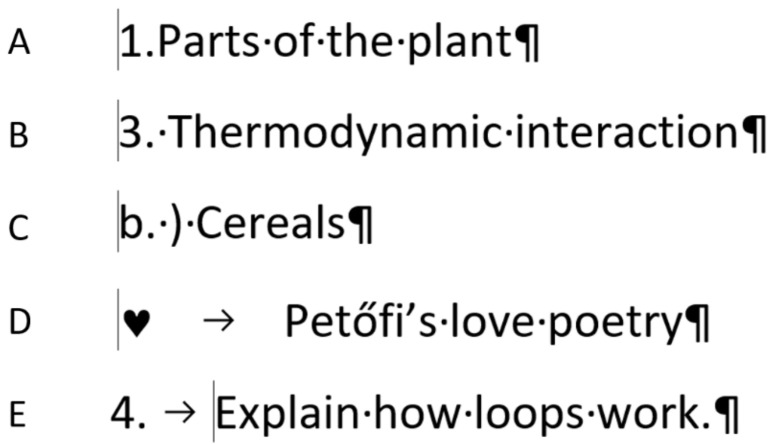
The translation of the five Hungarian expressions presented in the mini competence test.

The language of the samples does not play any role, but for better understanding, the translation is presented in Figure 8.

### 2.4. Formatted Automated Numbering

**Figure 9:**
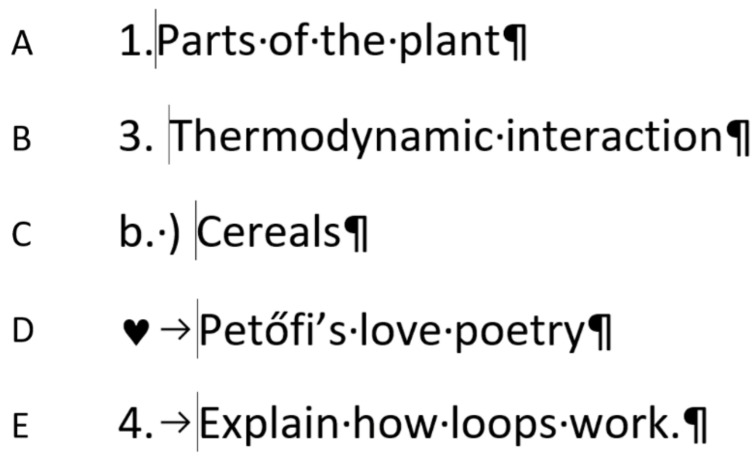
The properly formatted numbering of the samples of the mini competence test.

The position of the cursor can tell whether the numbering is manual or automated. However, formatting the automated numbering beyond the default settings requires further knowledge. Figure 9 presents how the samples can be solved with formatted automated numbering.

The comparison of Figure 7, Figure 8 and Figure 9 reveals that all the manual numbering can be replaced with formatted automated numbering which can be revealed by the leftmost position of the cursor.

Considering the semantics of automated numbering, four graphical variables would carry redundant information of the formatted automated numbering:

- The numbering character (number, letter, special character) (NC);
- The separator character (none, Space, Tabulator) (SC);
- The indentation of the paragraph;
- The position of the cursor.

However, to solve the task of the mini competence test, only the recognition of the position of the cursor is required.

## 3. Results

### 3.1. Results of the Test

In the first step of the evaluation of the mini competence task, the number of those students who answered the question correctly was calculated. Among the 8517 participants, 822 students (9.7%) only marked the correct answer (2 points). In the second phase, we checked the number of those students who, along with the correct answer, only marked one incorrect answer (1 point) (1363). All the other students (6332) got zero points (Table 1) [20].

The low percentage of two-point answers indicates that the students do not know that the position of the cursor must be checked. Along with the correct answer, one additional incorrect answer was accepted with one point. In these cases, additional information might lead the students to their selection. The possible variables based on the GUI are listed in Section 2.4. In the following phase of the evaluation, it was checked which variable had the strongest effect on the students’ choices.

**Figure 10:**
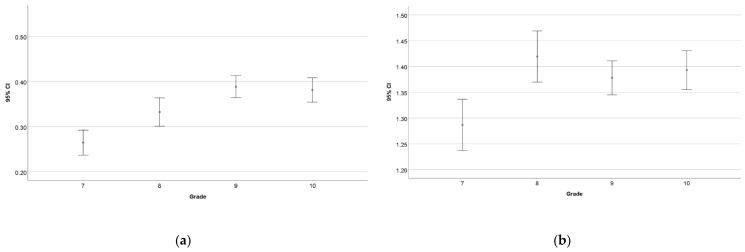
The differences between the average results of the different grades considering all the participating students (**a**) and only those whose result is greater than zero (**b**).

The result of grade 7 is significantly lower than those of the other grades, both including and leaving out the zero-point results. However, in all but one case, there are no significant differences between the grades. The only exception is between the results of grades 8 and 9 including the zero-point results (Figure 10).

**Figure 11:**
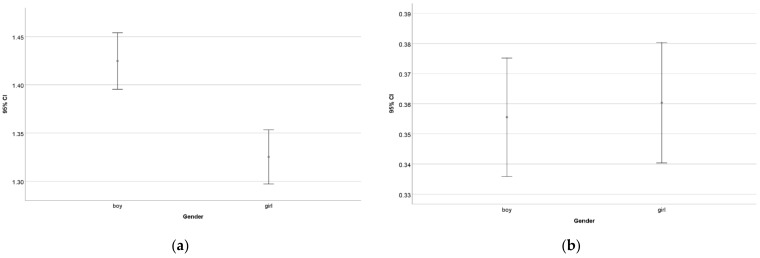
The differences between the average results of the different genders considering all students (**a**) and only those whose result is greater than zero (**b**).

The comparison of genders revealed no significant difference between boys and girls (*p* = 0.737) (Figure 11), including the zero-point results. However, when leaving out the zero-point results, a significant difference was found between boys and girls (*p* = 0.000). This result indicates that boys’ knowledge seems more stable than that of girls.

### 3.2. Clusters of the Students’ Results

**Figure 12:**
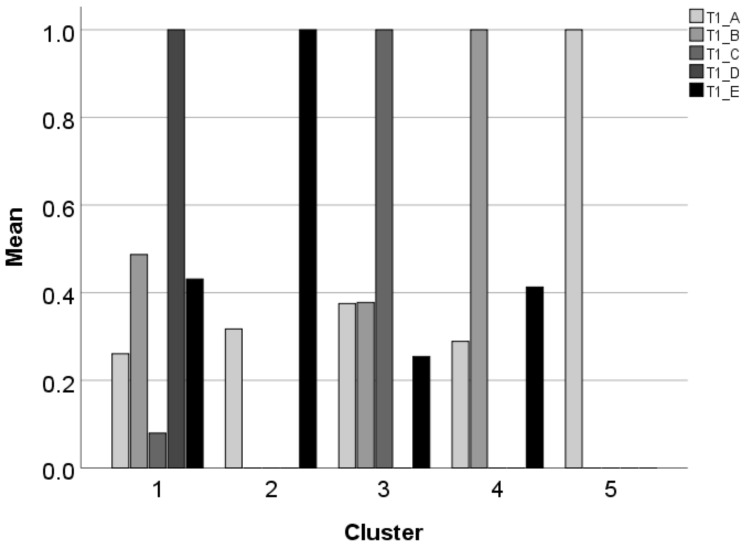
The clusters formed on the answers of the numbering task of the mini competence test.

To reveal the patterns of those students who marked at least one answer, a TwoStep Cluster analysis was carried out. The analysis found five clusters as shown in Figure 12 (the darkening colors indicate Answers A–D). The numbers of those in Clusters 1–5 are 2041, 1204, 845, 2280, and 939, respectively.

The clusters are formed on the rules presented in Table 2, Table 3, Table 4, Table 5 and Table 6. The Solutions columns list the different patterns where 1 means the marked options and 0 means the unmarked options. The Frequency and Percent columns indicate the number and percentage of students who selected a combination. It is notable that each cluster has a dominant answer and further answers are grouped around the selected one.

In Cluster 1 (Table 2), the dominant answer is D—the heart character as the numbering character followed by a fake Tabulator imitating automated numbering. This cluster is rather arbitrary with 16 different patterns identified, which means that the dominant answer does not provide enough information and does not play a leading role. The most frequent pattern is 01010 (B and D) where there is no connection between the two answers.

Two patterns belong to Cluster 2 (Table 3). The dominant one is 00001 (E), which is the correct answer, while the A and E combination forms the minor group. In this cluster, the position of the cursor plays the leading role. Furthermore, the combination of the number as the numbering character and of the separating character—the formatting Tabulator or nothing—seems to be the guideline.

Cluster 3 (Table 4) contains eight different patterns with C as the dominant one. This cluster has the second-greatest number of patterns. The most frequent patterns are 00100, 10100, and 01100 (C alone, C with A, and C with B). Answer C seems to contain misconception(s) which attract further wrong answers.

In Cluster 4 (Table 5), the dominant pattern is 01000 (B alone) with the combination of a number as the numbering character and a typed Space as the separator character. The second most popular pattern in this cluster is 01001 (B with E).

Cluster 5 (Table 6) has one pattern which is 10000 (A alone). In Answer A, there is a number as the numbering character without a separator character. Those students who selected this answer only recognized the number without knowing that, by default, the number is followed by a separator character.

The dominant answers of the clusters collected and presented in Table 7.

### 3.3. The Entropy of Automated Numbering

According to the samples presented in the test, the position of the cursor is enough to answer the question. Considering this information, the task can be described with an IID model. In this model, the probabilities of both the correct and the incorrect answers are *p* = 0.5. All the cursors positioned on the left are incorrect, and the one whose leftmost position is at the indentation is correct. This information can be coded with one single bit [25,26,27,28,29,30,31,32,33,34].

However, if the position of the cursor does not carry the information, redundant information would help to recall knowledge from long-term memory. According to the characteristics of the automated numbering, four variables were found (Section 2.4). The question was how the theory of information entropy would support the hypothesis. We wanted to find an objective measure of the number of bits that are needed to pass the information through the channel of the teaching–learning process.

Based on the dominant answer of each cluster and the four variables of the automated numbering, the groups in Table 8 were identified. To calculate the entropy of the automated numbering, for each group, the frequency and then the probability were calculated (Table 8) (Equation (1)).

(1) $$p_{1}+\cdots +p_{12}=\overset{12}{\underset{i=1}{\sum }}p_{i}=1$$

In the following step, the self-information of each group was calculated (Equation (2)).

(2) $$I_{k}\left(p\right)=-\log_{2}p_{k}$$

As the last step, based on the probability and self-information of the groups, the entropy of the automated numbering is calculated (Equation (3)).

(3) $$H\left(X\right)=\overset{12}{\underset{i=1}{\sum }}p_{k}\cdot I_{k}=-\overset{12}{\underset{i=1}{\sum }}p_{k}\cdot \log_{2}p_{k}=3.0961$$

It is found that the entropy is 3.0961. This means that at least three bits are needed to transfer the information of the GUI of the word processor to end-users. To gain the level of knowledge at which one bit—the position of the cursor—is enough to tell whether the numbering is automated, three bits of information must be put on the channel. This implies that, in the teaching–learning process, saying that automated numbering is nothing more than one click on one of the numbering buttons is not enough. Furthermore, the technical details of the command—the description of the tool: how to change the color, the shape, the size of the bullets, etc.—do not include the information needed to avoid manual numbering. The messages of the GUI must be taught and learned consciously. End-users must gain the knowledge that the developers of the word processors put on the GUI. The redundancy measured by the entropy would help teachers to build up their strategies and methods to provide enough information for end-users of different backgrounds to understand the semantics of automated numbering.

## 4. Discussion

The results of the mini competence test [20] reveal that most of the students in grades 7–10 cannot distinguish between manual and automated numbering in MS Word samples. They do not know that one bit of information put on the GUI—the position of the cursor—can be enough to decide which sample has manual or automated numbering. We must also note that, at the time of the testing, grade 10 was the last school year during which students study informatics in school in Hungary. This implies that digital students [35] leave school when only 10.8% of them in a multiple-choice question can click the correct answer, leaving space for hazardous answers [18]. This finding is in complete accordance with the results of Johnson who claimed that “A stand-alone computer skills course (taught by a school or by a computer dealer) may not be the best means to teach substantial knowledge of word processing; examples and practice will inevitably be simulated and artificial, and there will be little motivation to fully understand the applications” [1]. Wolfram came to the same conclusions by claiming that “…when major new machinery comes along—as computers have—it’s rather disorientating” [36,37].

These findings were supported by the analysis of Word documents and teaching, learning, tutorial, and testing materials carried out in advance of the mini competence test [20]. The supporting documents focus exclusively on tools, paying no attention to the information put on the GUI, the semantics of the commands of the word processor [11], or information that the non-printing characters carry [21,22,23,24]. Furthermore, course books, tutorials, and teachers—instead of paying attention to TPCK [38,39,40,41], developing computational thinking skills [42], real-world computer problem solving [43], the role of fast and slow thinking [10,44], and cognitive load theory [45]—primarily use decontextualized texts in the teaching–learning and testing [10,38,39,40,41,46] processes.

Focusing on the aims of the present paper—analyzing automated numbering—it was also found that even the supporting Word documents have manual numbering instead of the automated word-processing feature. This means that we are in great need of excellent teachers who are not only experienced but are aware of the essence of the teaching–learning process and methods that can be effectively and efficiently used in the digital era [10,47,48,49,50].

Considering all these related findings, the efficiency rate (e, Equation (4)) and the redundancy (R, Equation (5)) of the teaching–learning process was questioned. To calculate the *e* value, we take into consideration the fact that the higher the entropy of a random variable, the closer that random variable is to having all of its outcomes equally likely (H_max_).

(4) $$e=\frac{H\left(X\right)}{H_{max}}=\frac{3.0961}{3.7004}=0.8367$$

(5) R=1−e=1−0.8367=0.1633

The most interesting of all these findings is that, unlike in informatics (e.g., compression) and natural languages, redundancy plays a crucial role in the teaching–learning process [43,49,51]. The question is what the optimal redundancy rate is in the teaching–learning process of automated numbering [51]. It is found that the redundancy is between three and four bits of information for the automated numbering, which is in complete accordance with our intuitive measure.

However, we are convinced that the redundancy rate is different for the different features of word processing. Consequently, further analyses are required to reveal both the entropy and the redundancy of other word processing features and commands.

In general, we can conclude that, based on the results of the test and the calculated entropy and redundancy of automated numbering, we built up a method that would be generalized and used for other word-processing features. With this objective measuring method, we are able to identify how much information—beyond teaching the mere use of word-processing tools—must be put on the channel to pass all the information of the GUI to end-users.

## 5. Conclusions

Misconceptions based on the widely accepted and widespread rumor that word processing and word-processing tools are synonyms lead to inefficient and ineffective word-processing practices and ultimately erroneous word documents.

In the present study, the details of a method were described which includes the analysis of teaching, learning, tutorial, and testing sources, the collection and analysis of Word documents shared on the internet or in closed groups, the testing of grade 7–10 students’ knowledge in automated numbering, and the calculation of the entropy of automated numbering.

It was found that the information put on the GUI of the word procesor—MS Word, in this case—cannot reach either the tested students or the authors/editors/lectors of the analyzed documents. This implies that it is a false assumption that students are born with this ability and that teaching the tools of word processors is enough to learn word processing sufficiently to create properly edited and/or formatted digital texts [35,51,52,53,54,55]. The calculation of the entropy reveals that at least three times more information should be put on the channel—i.e., the teaching–learning process—to gain one-bit knowledge.

## Figures and Tables

**Table 1:** The percentage of students who got 2, 1, and 0 points in the numbering task of the mini competence test.

Title 1	Total	Grade 7	Grade 8	Grade 9	Grade 10
2 points	9.7	5.9	9.8	10.7	10.8
1 point	16.0	14.7	13.6	17.5	16.6
0 points	74.3	79.4	76.6	71.8	72.6

**Table 2:** The combinations of answers in Cluster 1.

Solutions	Frequency	Percent
00010	309	15.1
00011	301	14.7
00110	29	1.4
00111	8	0.4
01010	451	22.1
01011	373	18.3
01110	20	1.0
01111	18	0.9
10010	265	13.0
10011	102	5.0
10110	30	1.5
10111	3	0.1
11010	51	2.5
11011	27	1.3
11110	6	0.3
11111	48	2.4
Total	2041	100.0

**Table 3:** The combinations of answers in Cluster 2.

Solutions	Frequency	Percent
00001	822	68.3
10001	382	31.7
Total	1204	100.0

**Table 4:** The combinations of answers in Cluster 3.

Solutions	Frequency	Percent
00100	268	31.7
00101	48	5.7
01100	150	17.8
01101	62	7.3
10100	157	18.6
10101	53	6.3
11100	55	6.5
11101	52	6.2
Total	845	100.0

**Table 5:** The combinations of answers in Cluster 4.

Solutions	Frequency	Percent
01000	989	43.4
01001	632	27.7
11000	350	15.4
11001	309	13.6
Total	2280	100.0

**Table 6:** The pattern in Cluster 5.

Solutions	Frequency	Percent
10000	939	100.0
Total	939	100.0

**Table 7:** The dominant answers of the clusters.

Cluster	Dominant Answer	Pattern
1	D	???1?
2	E	?0001
3	C	??10?
4	B	?100?
5	A	10000

**Table 8:** The groups of answers formed on the dominant answers of the clusters and the three independent variables of the automated numbering (NC stands for Numbering Character, and SC stands for the Separator Character between the number and the typed text).

Group	Pattern	Frequency	Probability	Identifiers
A	10000	939	0.13	dominant = Cluster 5
B	01000	989	0.14	dominant ∈ Cluster 4
C	00100	268	0.04	dominant ∈ Cluster 3
D	00010	309	0.04	dominant ∈ Cluster 1
E	00001	822	0.11	dominant ∈ Cluster 2
ABE	11001	309	0.04	number as NC ∈ Cluster 4
BE	01001	632	0.09	number as NC followed by a SC ∈ Cluster 4
DE	00011	301	0.04	Tabulator as SC ∈ Cluster 1
BC	01100	150	0.02	Space as SC ∈ Cluster 3
BCE	01101	62	0.01	number or letter as NC followed by a SC∈ Cluster 3
AE	10001	382	0.05	number as NC without SC = Cluster 2
Other		2146	0.29	
